# Single versus dual blockade of the renin-angiotensin system in patients with IgA nephropathy

**DOI:** 10.1007/s40620-020-00836-8

**Published:** 2020-08-27

**Authors:** David Paul Lennartz, Claudia Seikrit, Stephanie Wied, Christina Fitzner, Frank Eitner, Ralf-Dieter Hilgers, Thomas Rauen, Jürgen Floege

**Affiliations:** 1 Division of Nephrology and Clinical Immunology, RWTH Aachen University, Pauwelsstr. 30, 52057 Aachen, Germany; 2 Department of Medical Statistics, RWTH Aachen University, Aachen, Germany; 3 Kidney Diseases Research, Bayer AG, Wuppertal, Germany

**Keywords:** Renin-angiotensin system, RAS system, RAS blockers, IgA nephropathy, STOP-IgAN

## Abstract

**Background:**

Inhibitors of the renin-angiotensin system (RAS) are cornerstones of supportive therapy in patients with IgA nephropathy (IgAN). We analyzed the effects of single versus dual RAS blockaQueryde during our randomized STOP-IgAN trial.

**Methods:**

STOP-IgAN participants with available successive information on their RAS treatment regimen and renal outcomes during the randomized 3-year trial phase were stratified post hoc into two groups, i.e. patients under continuous single or dual RAS blocker therapy over the entire 3 years of the trial phase. Primary and secondary STOP-IgAN trial endpoints, i.e. frequencies of full clinical remission, eGFR-loss ≥ 15 and ≥ 30 ml/min/1.73 m^2^ and ESRD onset, were analyzed by logistic regression and linear mixed effects models.

**Results:**

Among the 112 patients included in the present analysis, 82 (73%) were maintained on single and 30 (27%) on dual RAS inhibitor therapy throughout the trial. Neither RAS blocker strategy significantly affected full clinical remission, eGFR-loss rates, onset of ESRD. Proteinuria moderately increased in patients under dual RAS blockade by 0.1 g/g creatinine during the 3-year trial phase. This was particularly evident in patients without additional immunosuppression during the randomized trial phase, where proteinuria increased by 0.2 g/g creatinine in the dual RAS blockade group. In contrast, proteinuria decreased in patients under single RAS blocker therapy by 0.3 g/g creatinine. The course of eGFR remained stable and did not differ between the RAS treatment strategies.

**Conclusion:**

In the STOP-IgAN cohort, neither RAS blocker regimen altered renal outcomes. Patients on dual RAS blockade even exhibited higher proteinuria over the 3-year trial phase.

## INTRODUCTION

IgA nephropathy (IgAN) is the most common type of glomerulonephritis in the western world [1]. It usually runs a chronic, often slowly progressive course and there is wide consensus that blood pressure control and other measures, collectively termed “supportive care”, constitute a mainstay of therapy [2, 3] before immunosuppressive strategies may be considered [4].

Blood pressure increases very early in IgAN patients and even seemingly normotensive patients have higher blood pressures than matched healthy controls and exhibit subtle cardiac changes [5]. In addition, there is considerable evidence of early activation of the renin-angiotensin system (RAS) in the kidneys of IgAN patients [6, 7], and this has been related to the pathogenesis of tubulointerstitial damage [8]. Combined with the well-established antiproteinuric action of RAS blockers, this lays the basis for a strong recommendation to initiate and up-titrate ACE-inhibitors or angiotensin-receptor blockers (ARB) in all proteinuric IgAN patients at risk for progressive renal disease [2]. This recommendation is backed by randomized controlled clinical trials in IgAN showing better renal outcome in patients treated with enalapril versus non-RAS blocker antihypertensives [9] or benazepril versus placebo [10].

Already 18–20 years ago small clinical trials in IgAN patients reported that the combination of losartan with an ACE-inhibitor exerts additive antiproteinuric effects that were independent of achieved blood pressures [11, 12]. This was then confirmed and extended to various other glomerular diseases [13–15]. However, subsequent major clinical trials focusing on renal and/or cardiovascular outcome in diabetic patients failed to detect a benefit from combined ARB – ACE inhibitor therapy in diabetic kidney disease but rather noted a higher risk of acute kidney injury and hyperkalemia with the combination as compared to the single substances [16, 17]. The higher prevalence of such adverse events may relate to a too rapid up-titration to maximal doses with consecutive extra- and intrarenal vascular changes in diabetics that result in manifest or functional renal artery stenosis and thus a pronounced fall in renal perfusion with dual RAS blockade and subsequent acute renal injury. It is currently unknown whether this problem also occurs in primary glomerular diseases such as IgAN, in particular since it usually manifests in younger adults with relatively low atherosclerotic burden. In the present analysis we aimed to analyze whether renal endpoints including courses of renal function and proteinuria were affected by single or dual RAS blockade in patients that were included in the randomized controlled STOP-IgAN trial [3].

## METHODS

### Main STOP-IgAN trial

The STOP-IgAN trial was a multicenter, open label, randomized, controlled study that recruited 337 patients with biopsy-proven IgA nephropathy between February 2008 and October 2011. Protocol and results from the original trial have been published [3, 18]. Briefly, eligible patients entered a 6-month run-in phase with comprehensive optimization of supportive treatment strategies including antihypertensive therapy with RAS blockers targeting a blood pressure below 125/75 mmHg. Additional measures comprised dietary counseling, cholesterol lowering, education to quit smoking, and avoidance of nonsteroidal anti-inflammatory drugs and other nephrotoxins. Upon completion of the run-in phase, 162 patients with persistent proteinuria > 0.75 g/day, but less than 3.5 g/day, despite optimized supportive care were then randomized into the following 3-year trial phase and were assigned to either continue on supportive therapy alone or to receive additional immunosuppression. Co-primary hierarchically ordered endpoints of the STOP-IgAN trial comprised achievement of full clinical remission (i.e. proteinuria below 0.2 g/g and eGFR-loss less than 5 ml/min/1.73 m^2^ at the end of the 3-year trial phase) and eGFR-loss ≥15 ml/min/1.73m^2^ over the trial phase. Rates of eGFR-loss ≥ 30 ml/min/1.73m^2^ and onset of ESRD during the trial phase were captured as secondary endpoints.

### Study design and participants

Among the entire cohort of 162 randomized STOP-IgAN participants, available endpoint information and a complete data set on RAS blocker treatment (i.e. at the time of randomization as well as 12, 24 and 36 months after randomization) were available for 112 patients. These individuals were classified post hoc based on their individual RAS blocker treatment regimen into patients on continuous single or dual RAS blocker therapy. A distinction between different ACE inhibitor or ARB substances was not made. Among the originally randomized cohort, 50 patients were not included in the present analysis due to missing information on RAS (n = 32) treatment at any of the indicated time-points or since RAS treatment strategy had been interrupted (n = 7) or changed from single to dual RAS blockade (n = 2) or vice versa (n = 9). Systolic and diastolic blood pressure was recorded as office blood pressure measured at the time of randomization as well as 12, 24 and 36 months after randomization. Detailed information on antihypertensive treatment was captured at randomization and upon completion of the 3-year trial phase.

### Serum aldosterone assessment

Serum aldosterone levels were measured in samples obtained at the end of the trial phase using the Parameter® Aldosterone Assay (R&D Systems, Biotechne, Abingdon, UK) following the manufacturer's instructions in a threefold dilution.

### Statistical analyses

All statistical analyses were performed with SAS 9.4 Software (SAS Institute Inc., Cary, NC, USA). Data are presented as mean ± standard deviations for continuous variables and as counts and percentages for binary variables. To test the occurrence of the binary primary and secondary endpoints of the main STOP-IgAN trial in the two RAS blocker treatment groups, we applied a logistic regression model (proc LOGISTIC in SAS) adjusting for age, baseline GFR (≥ 60 vs. < 60 ml/min/1.73 m^2^) and proteinuria (< 1.5 vs. ≥ 1.5 g/day) as well as the treatment arm (supportive care vs. supportive care plus immunosuppression) during the 3-year trial phase. Due to quasi complete separation in the analyses of the events “eGFR-loss ≥ 30 ml/min/1.73m^2^ “ and “ESRD” the *Firth* correction was applied.

The course of proteinuria over the 3-year trial phase was analyzed using a linear mixed effects model with random intercept and slope (proc MIXED in SAS). As fixed effects we modeled age, baseline GFR (≥ 60 vs. < 60 ml/min/1.73 m2) and proteinuria (< 1.5 vs. ≥ 1.5 g/day), treatment and RAS blocker treatment. We used the variance components covariance structure and adjusted the degrees of freedom by the “between-and-within” method. The residual plots were examined visually to assess the model fit and extreme outliers were excluded based on the restricted likelihood distance. Although this is an explorative evaluation, p values ≤ 0.05 were categorized as significant.

## RESULTS

### Intervention groups and baseline characteristics

**Fig. 1:**
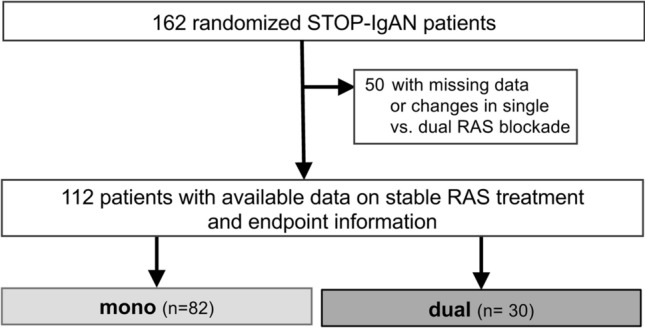
Flowchart of analyzed patients

A complete data set on renal function, proteinuria, study endpoints and RAS treatment over the entire 3.5 years was available for 112 patients (i.e. 69% of the 162 patients who completed the original STOP-IgAN trial). Among these patients, 82 (73%) stably received single RAS blocker therapy and 30 (27%) patients received continuous dual RAS blocker therapy during the trial phase (Fig. 1). Patients’ demographic and clinical characteristics at enrollment into the trial are given in Table 1. Treatment allocation to either continue supportive care or receive additional immunosuppression during the subsequent 3-year trial phase was comparable between the two RAS blocker intervention groups. Patients on dual RAS blockade were equally distributed across the participating centers.

**Table 1:** Baseline characteristics of trial participants at randomization based on stratification of RAS blockade regimen during the STOP-IgAN trial

Subgroup	Single	Dual	Excluded
	RAS blockade	RAS blockade	Patients
	(n = 82)	(n = 30)	(n = 50)
Female sex (%)	28	13	16
Smoker (%)	18	21	13
Age (years)	45.5 ± 12.2	44.5 ± 12.8	43.8 ± 13.1
Body mass index (kg/m^2^)	27.8 ± 5.3	29.8 ± 6.2	26.8 ± 4.1
Blood pressure (mmHg)	126 ± 14	134 ± 18	132 ± 15
systolic	77 ± 11	81 ± 11	83 ± 12
diastolic			
GFR (ml/min)	76.8 ± 32.6	79.1 ± 33.6	74.4 ± 36.5
Urinary protein excretion (g/d)	1.6 ± 0.8	1.7 ± 0.8	1.8 ± 0.7
Urinary protein-to-creatinine ratio (g/g)	1.0 ± 0.9	1.0 ± 0.6	1.1 ± 0.6
Patients randomized to supportive care in the trial phase (%)	52	53	42

Table 1 also shows that the 50 patients who were excluded from the present analysis exhibited comparable baseline characteristics to those of the included patients.

### Renal outcomes in the different RAS intervention groups

**Fig. 2:**
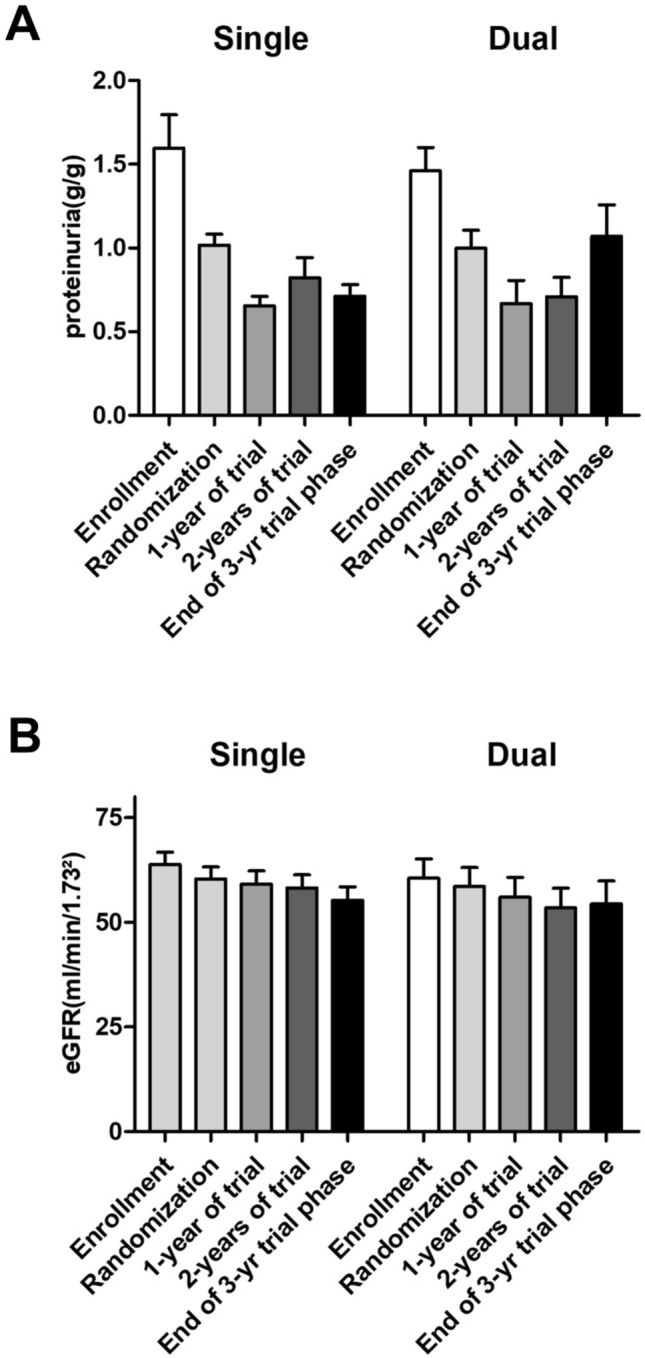
Urinary protein excretion (**a**) and eGFR (**b**) at enrollment, at randomization (i.e. at the end of the 6-month run-in phase) and at the end of the 3-year trial phase in patients under single versus dual RAS blockade

In both RAS blocker intervention groups, the occurrence of any of the primary and secondary renal endpoints at the end of the subsequent 3-year trial phase, i.e. achievement of full clinical remission, eGFR-loss of ≥ 15 or ≥ 30 ml/min/1.73m^2^ and onset of ESRD did not differ significantly (Table 2). Patients on stable dual RAS blocker therapy moderately increased their proteinuria by 0.1 g/g, whereas patients on stable single RAS blocker therapy significantly decreased their proteinuria by 0.3 g/g over the study period (Fig. 2a). Multivariate analyses demonstrated that both the RAS strategy (p = 0.011) as well as treatment allocation to either supportive care alone or additional immunosuppression (p = 0.039) significantly affected the course of proteinuria over the trial phase (Table 3). A further sensitivity analysis that also included patients for whom RAS blocker information was available at only two out of three time points (i.e. 12, 24 and 36 months upon randomization) contributed 20 additional patients to the analysis (with only 30 remaining STOP-IgAN patients without such information) and entirely confirmed the primary analysis (data not shown). Estimated GFR and proteinuria courses separated by RAS therapy and treatment allocation to either supportive care alone or additional immunosuppression are given in Table 4. A decrease in proteinuria by 0.5 g/g over the trial phase was observed in patients under single RAS inhibition and additional immunosuppression. There was an increase in proteinuria in patients receiving dual RAS blockade, but no additional immunosuppression. Estimated GFRs remained almost stable over the entire STOP-IgAN trial in both RAS intervention groups between enrollment and end of the trial (Fig. 2b).

**Table 2:** Primary and secondary trial endpoints of analyzed patients in the RAS treatment groups

Subgroup	Single RAS blockade		Dual RAS blockade		*p* value (adjusted)
	N	mean ± SD or no. (%)	N	mean ± SD or no. (%)	
In full clinical remission	78	12 (15)	30	4 (13)	0.920
eGFR-drop ≥ 15 ml/min/1.73m^2^	82	15 (18)	30	5 (17)	0.692
eGFR-drop ≥ 30 ml/min/1.73m^2^	82	2 (2)	30	2 (7)	0.405
ESRD onset	82	1 (6)	30	0 (0)	0.813

**Table 3:** Annual change in proteinuria since randomization (linear mixed model)

Annual change in proteinuria (g/g)			
Predictor	Estimate	95% CI	*p* value
RAS treatment strategy	− 0.163	− 0.287–0.039	0.011
Age	0.001	− 0.004–0.005	0.765
Time	− 0.024	− 0.065–0.017	0.254
Baseline GFR ≥ or (< 60 ml/min/1.73 m^2^)	− 0.267	− 0.388–0.146	< 0.0001
Baseline proteinuria (< 1.5 or ≥ 1.5 g/day)	− 0.406	− 0.518–0.294	< 0.0001
Treatment during trial phase (SUP vs. IMM)	0.115	0.006–0.224	0.039

**Table 4:** Course of renal function and proteinuria, depending on continuous RAS treatment strategy over the randomized 3-year trial phase

		eGFR (ml/min/1.73 m^2^)			Urinary protein-to-creatinine ratio (g/g)		
		*N*	At randomization	End of trial phase	*N*	At randomization	End of trial phase
Single RAS blockade during trial phase	Supportive care	43	58.2 ± 25.7	51.1 ± 29.5	41	1.0 ± 0.5	0.8 ± 0.6
	Additional immunosuppression	39	62.7 ± 27.1	59.9 ± 28.0	37	1.1 ± 0.6	0.6 ± 0.6
Dual RAS blockade during trial phase	Supportive care	16	59.7 ± 29.8	55.3 ± 38.0	16	0.9 ± 0.5	1.1 ± 0.8
	Additional immunosuppression	14	57.4 ± 18.3	53.4 ± 18.6	14	1.2 ± 0.7	1.0 ± 1.2

### Blood pressure changes and antihypertensive management during the trial phase

At the time of randomization, mean systolic blood pressure was higher in patients under dual RAS blockade as compared to those under single RAS therapy (Table 5). Yet, mean diastolic blood pressure levels did not differ. Over the subsequent 3-year trial phase, blood pressure levels remained stable in both arms without obvious differences between the two RAS intervention groups. At randomization, as well as at the end of the trial phase, a higher number of patients under dual RAS blockade more commonly received more than 3 antihypertensive agents than those under single RAS blockade. However, there were no obvious differences in the numbers of antihypertensive agents being increased, decreased or kept on a stable level between the two RAS intervention arms (Table 5). The number of patients under maximum allowed ACE inhibitor therapy according to prescribing information remained unchanged under dual RAS blockade over the study period. By contrast, ARB dosage had been maximized at the end of the 3-year trial phase in four additional patients on single RAS blockade compared to two patients on a dual RAS blocker therapy strategy (Table 5).

**Table 5:** Course of blood pressure (bp) and antihypertensive treatment in the RAS treatment groups

Subgroup	Single RAS blockade (*n* = 112)		Dual RAS blockade (*n* = 30)	
	*N*	Mean ± SD or no. (%)	*N*	Mean ± SD or no. (%)
*Blood pressure (mmHg)*				
At randomization				
Systolic	80	125.5 ± 13.7	29	134.1 ± 18.5
Diastolic		77.5 ± 10.8		80.7 ± 10.7
12 months after randomization				
Systolic	81	127.1 ± 14.2	30	128.8 ± 10.7
Diastolic		78.8 ± 9.1		79 ± 8.6
24 months after randomization Systolic Diastolic	82	129.1 ± 15.6 81.3 ± 9.6	29	133.4 ± 17.5 79.5 ± 10.1
36 months after randomization Systolic Diastolic	81	127.7 ± 16.2 80.7 ± 12.6	30	131.1 ± 17.9 81.8 ± 10.6
*Antihypertensive agents No. (% of subgroup)*				
At randomization 1 bp medication 2 bp medications 3 bp medications > 3 bp medications	82	21 (26) 23 (28) 13 (15.9) 25 (30.5)	30	0 (0) 10 (23.3) 4 (16.7) 16 (53.3)
36 months after randomization 1 bp medication 2 bp medications 3 bp medications > 3 bp medications	82	15 (18.3) 21 (25.6) 12 (14.6) 34 (41.5)	30	0 (0) 7 (23.3) 5 (16.7) 18 (60.0)
*Adjustment of antihypertensive drugs over the 3-year trial*				
Increased	82	27 (32.9)	30	11 (36.7)
Unchanged	82	44 (53.7)	30	16 (53.3)
Decreased	82	11 (13.4)	30	3 (10)
*Maximum ACE dosage*				
At randomization	82	30 (36.6)	30	17 (56.7)
36 months after randomization	82	23 (28.1)	30	17 (56.7)
*Maximum ARB dosage*				
At randomization	82	20 (24.4)	30	12 (40)
36 months after randomization	82	24 (29.3)	30	14 (46.7)

### Serum aldosterone levels during the trial phase

**Fig. 3:**
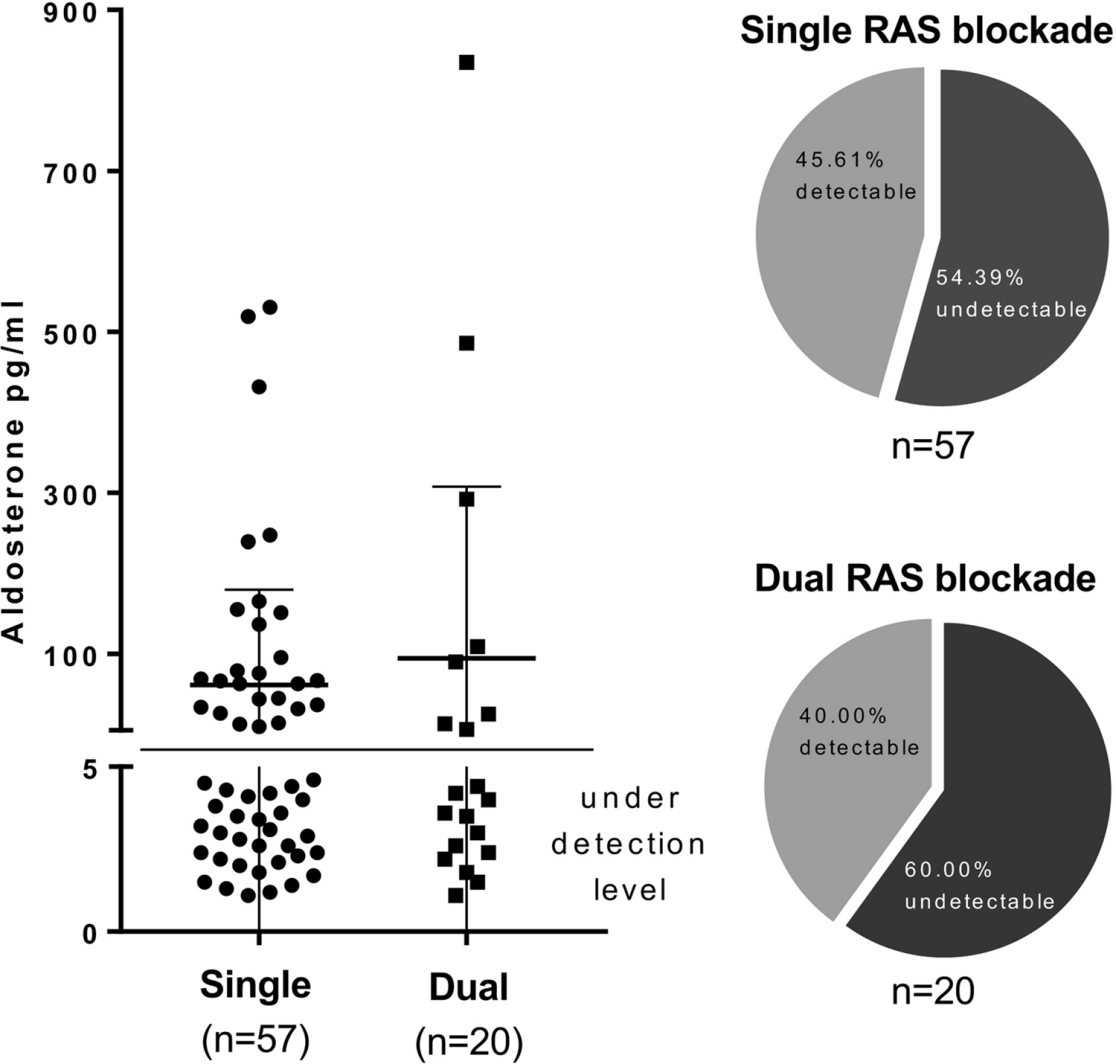
Aldosterone measurements in available serum samples obtained at the end of the trial phase according to continuous single or dual RAS blocker therapy during the randomized 3-year trial phase

To assess potential mechanisms accounting for the increase in proteinuria with dual RAS blockade, we measured aldosterone levels in patients with available serum samples obtained at the end of the trial (in 57 patients under single and 20 patients under dual RAS blockade). Aldosterone levels were suppressed, i.e. below the detection limit (i.e. 22 pg/ml), in 56% of the analyzed samples and detectable values did not differ between patients under continuous single or dual RAS blockade over the trial phase (Fig. 3).

## DISCUSSION

There is no doubt that inhibition of the RAS system is the most widely accepted treatment to achieve blood pressure and proteinuria targets in IgAN patients and that it is one of the major pillars of supportive treatment in these patients [2, 9, 19–21]. Years ago, smaller studies, mostly in patients with diabetes, suggested favorable anti-proteinuric effects from an ACE inhibitor/ARB combination [22] and dual RAS blockade was also applied to other patients. Yet, further evidence from clinical trials and meta-analyses demonstrated that dual RAS blockade provoked serious side effects including severe hypotension, hyperkalemia and renal failure in diabetic patients [17, 23, 24]. The randomized ONTARGET study did not show improved renal and cardiovascular outcomes under a fixed and rapid up-titration of dual RAS blockade over a run-in period of 3–4 weeks in patients at risk for cardiovascular disease. ONTARGET participants under dual RAS blockade even exhibited an increased rate of acute renal failure in this cohort [16]. Other randomized trials, such as LIRICO, VALID and PREPARE-2 comparing single with dual RAS blockade did not show deterioration of renal function in the dual approach but failed to detect renal benefits in diabetic and non-diabetic patients [25–27]. Current KDIGO guidelines do not recommend the use of dual RAS blockade in proteinuric IgAN patients since there is insufficient evidence to prove renal benefits from such strategy [2]. In 2014, the *EMA´s Committee for Medicinal Products for Human Use* even endorsed restrictions for the use of a dual RAS blockade [28].

Despite the above, the use of dual RAS blockade in IgAN and other glomerulonephritides is a matter of ongoing debate. In particular, it has been argued that patients with IgAN and many other types of glomerulonephritis are often younger adults and do not exhibit widespread cardiovascular disease, in particular stenosing atherosclerotic vessel damage, which in turn might predispose patients to acute kidney injury in the case of dual RAS blockade.

The STOP IgAN trial was conducted from 2008 to 2011 at 32 German centers. About 27% of all patients from the trial phase received dual RAS blocker therapy throughout the trial. In the present post hoc analysis, we investigated the association of dual versus single RAS blocker strategy on renal outcome parameters. None of the primary and secondary STOP-IgAN outcome measures, such as full clinical remission, eGFR-drop rates and ESRD occurrence, were affected by the RAS blocker regimen. Patients under dual RAS blockade even exhibited an increase in mean proteinuria to the end of the trial phase as compared to patients under single RAS blockade, in whom proteinuria decreased. Mean eGFR in both groups remained stable over the entire trial.

Reasons to initiate dual RAS blockade in IgAN patients at risk for a progressive disease course might be attempts to lower blood pressure as well as proteinuria and thus renal risk. However, at least with regard to proteinuria, our data rather disprove a long-lasting anti-proteinuric effect of dual RAS blockade. Multivariate analyses demonstrated that the RAS treatment strategy during our trial phase (i.e. single vs. dual inhibition) as well as treatment allocation to supportive care alone or additional immunosuppression, significantly affected the course of proteinuria. Patients under single RAS blockade and additional immunosuppression exhibited decreased proteinuria over the trial phase, whereas those under dual RAS blockade but with no additional immunosuppression had increased proteinuria. While the former could point to an improved long-term outcome, our very recent analysis of renal outcomes of the STOP-IgAN cohort after a mean follow-up of 7.4 years did not indicate any lasting benefit of immunosuppression on an endpoint composed of death, dialysis and 40% eGFR loss [29].

The observed increase in proteinuria in patients under dual RAS blockade at the end of the 3-year trial phase was unexpected and cannot be concisely explained based on the available data and our analysis strategy. Of note, systolic blood pressure at randomization tended to be higher in the “dual RAS blockade” group, however, during the randomized trial phase the courses of blood pressure levels were comparable between the two arms. In addition, we did not find elevated serum aldosterone levels in patients under dual RAS blockade as compared to those under single therapy. Increased serum aldosterone has been described for proteinuric patients with diabetic nephropathy under single ARB therapy [30]. Currently, mineralocorticoid receptor antagonists are primarily recommended as a second-line alternative in patients who do not tolerate ACEi or ARBs. Alternatively, we cannot exclude a possible selection bias. Despite attempts to account for this statistically, we cannot completely exclude that the increased proteinuria in patients under dual RAS blockade was due to slightly higher average blood pressures (Table 5) or higher IgAN disease activity. However, at least the baseline proteinuria levels were similar in both groups suggesting that there was no systematic selection bias.

Our study is limited by its post hoc character and the small sample size. Of note, the original trial was not powered to detect differences in proteinuria between patients under single and dual RAS blocker treatment. Third, in the present analysis we experienced a significant loss of 50 originally randomized patients who were not included in the present post hoc analysis due to missing data or, more importantly, due to changes in RAS therapy strategy. However, the excluded patients from the present analysis had comparable baseline characteristics versus those who had been included. Lastly, we cannot exclude a selection bias since decisions on single or dual RAS treatment regimen were based upon the physician’s discretion in the clinical routine and did not follow a protocol-defined algorithm. At the time of enrollment, patients under dual RAS blockade tended to have higher mean systolic blood pressure, received more antihypertensives and tended to have an increased body mass index than those under single RAS inhibition suggesting that these patients per se exhibited features of an unfavorable natural course of their IgAN. However, in the present secondary analysis, none of these differences achieved statistical significance.

Our results from this secondary analysis of a STOP-IgAN subcohort shed new light on the role of dual RAS inhibition in patients with IgAN, yet we cannot exclude some degree of selection bias. Consistent with numerous prior RCTs, predominantly in diabetic nephropathy, we failed to obtain evidence that dual RAS blockade exerted beneficial renal effects in IgAN. Rather, patients on this regimen even exhibited higher proteinuria at the end of the randomized 3-year trial phase, independent of the STOP IgAN treatment allocation to supportive care alone or additional immunosuppression. Our data thereby support the current approach backed by KDIGO guidelines (www.kdigo.org) to up-titrate individual RAS blockers to maximally allowed or tolerated dosages rather than combining them.
